# Performance comparison of four types of target enrichment baits for exome DNA sequencing

**DOI:** 10.1186/s41065-021-00171-3

**Published:** 2021-02-17

**Authors:** Juan Zhou, Mancang Zhang, Xiaoqi Li, Zhuo Wang, Dun Pan, Yongyong Shi

**Affiliations:** Bio-X Institutes, Key Laboratory for the Genetics of Developmental and Neuropsychiatric Disorders (Ministry of Education), Collaborative Innovation Center for Brain Science, Shanghai Jiao Tong University, Shanghai, 200030 People’s Republic of China

**Keywords:** Next-generation sequencing, Exome capture efficiency, Bait type, Coverage, GC bias, SNPs and Indels detection

## Abstract

**Background:**

Next-generation sequencing technology is developing rapidly and target capture sequencing has become an important technique. Several different platforms for library preparation and target capture with different bait types respectively are commercially available. Here we compare the performance of the four platforms with different bait types to find out their advantages and limitations. The purpose of this study is to help investigators and clinicians select the appropriate platform for their particular application and lay the foundation for the development of a better target capture platform for next-generation sequencing.

**Results:**

We formulate capture efficiency as a novel parameter that can be used to better evaluations of specificity and coverage depth among the different capture platforms. Target coverage, capture efficiency, GC bias, AT Dropout, sensitivity in single nucleotide polymorphisms, small insertions and deletions detection, and the feature of each platform were compared for low input samples. In general, all platforms perform well and small differences among them are revealed. In our results, RNA baits have stronger binding power than DNA baits, and with ultra deep sequencing, double stranded RNA baits perform better than single stranded RNA baits in all aspects. DNA baits got better performance in the region with high GC content and RNA baits got lower AT dropout suggesting that the binding power is different between DNA and RNA baits to genome regions with different characteristics.

**Conclusions:**

The platforms with double stranded RNA baits have the most balanced capture performance. Our results show the key differences in performance among the four updated platforms with four different bait types. The better performance of double stranded RNA bait with ultra deep sequencing suggests that it may improve the sensitivity of ultra low frequent mutation detection. In addition, we further propose that the mixed baits of double stranded RNA and single stranded DNA may improve target capture performance.

**Supplementary Information:**

The online version contains supplementary material available at 10.1186/s41065-021-00171-3.

## Background

Next-generation sequencing technology is one of the most important tools for genomic research today because of its high throughput, sensitivity and specificity. The target capture sequencing which only focuses on the functional regions in the genome such as whole-exome sequencing, with the advantages of relatively low cost, available high depth and coverage, and easy dataset to manage [1], has become a routine technique in basic research and clinical diagnostics. Now, there are several alternative commercial platforms for target capture with different bait types, single and double stranded DNA/RNA baits respectively.

Several articles have compared the whole-exome capture performances of these platforms. Chilamakuri et al. compared the performance of four commercial whole-exome platforms: Agilent’s SureSelect XT2 Human All Exon v4.0, NimbleGen’s SeqCap EZ v3.0, Illumina’s Nextera Rapid Capture Exome and TruSeq Exome Enrichment kit. They found that Illumina covered more bases in coding and untranslated regions [1]. Clark et al. reported that the Nimblegen needed the least number of reads to sensitively detect small variants. Agilent and Illumina detected a greater total number of variants with additional data [2]. In another research, the performance comparison for the four commercial platforms, Roche/NimbleGen’s SeqCap EZ Human Exome Library v3.0, Illumina’s Nextera Rapid Capture Exome (v1.2), Agilent’s SureSelect XT Human All Exon v5 and Agilent’s SureSelect QXT was conducted and Agilent SureSelect XT Human All Exon v5 showed the highest target enrichment efficiency and the best SNPs (single nucleotide polymorphisms) and short Indels (small insertions and deletions) detection sensitivity in coding regions with the least amount of sequencing data [3]. The performance comparison between the Agilent v6 exome and NimbleGen’s MedExome showed that Agilent v6 exome was the better choice to identify novel disease-associated genes as well as NimbleGen’s MedExome was better to detect the relevant, disease-causing mutations [4]. Recently, Iadarola B. et al. assessed the performance of short (~ 200 bp), medium (~ 350 bp) and long (~ 500 bp) DNA fragments on four major commercial exome enrichment platforms produced by IDT, Roche, Agilent and Twist, and they found that longer DNA fragments achieved a higher genotypability [5].

However, substantial updates have been released for each of these whole-exome capture platforms and several new brands of commercial kits were available over the past few years. At present, there is neither research comparing the performance of these updated or new whole-exome platforms with different bait types, nor research comparing the performance of the custom panel from different platforms with ultra deep sequencing. In order to help investigators and clinicians select the appropriate platform for their particular application and lay the foundation for the development of a better target capture platform for next-generation sequencing, we select a typical and the most recent exome enrichment platform for each bait types and present insights into the performance of them. Among them, the bait type of SureSelect Human All Exon v7 (Agilent Technologies) is single stranded RNA, xGEN Exome Research Panel v1.0 (Integrated DNA Technologies) is single stranded DNA, Human Core Exome (Twist Bioscience) is double stranded DNA and QuarXeq Human All Exon Probes 1.0 (Dynegen Bioscience) is double stranded RNA. And we further compare the performance with ultra deep sequencing of two custom capture panels which covered the same genome region but with different bait types, single and double stranded RNA respectively.

## Results

### Features comparison of the four whole-exome capture platforms

**Fig. 1:**
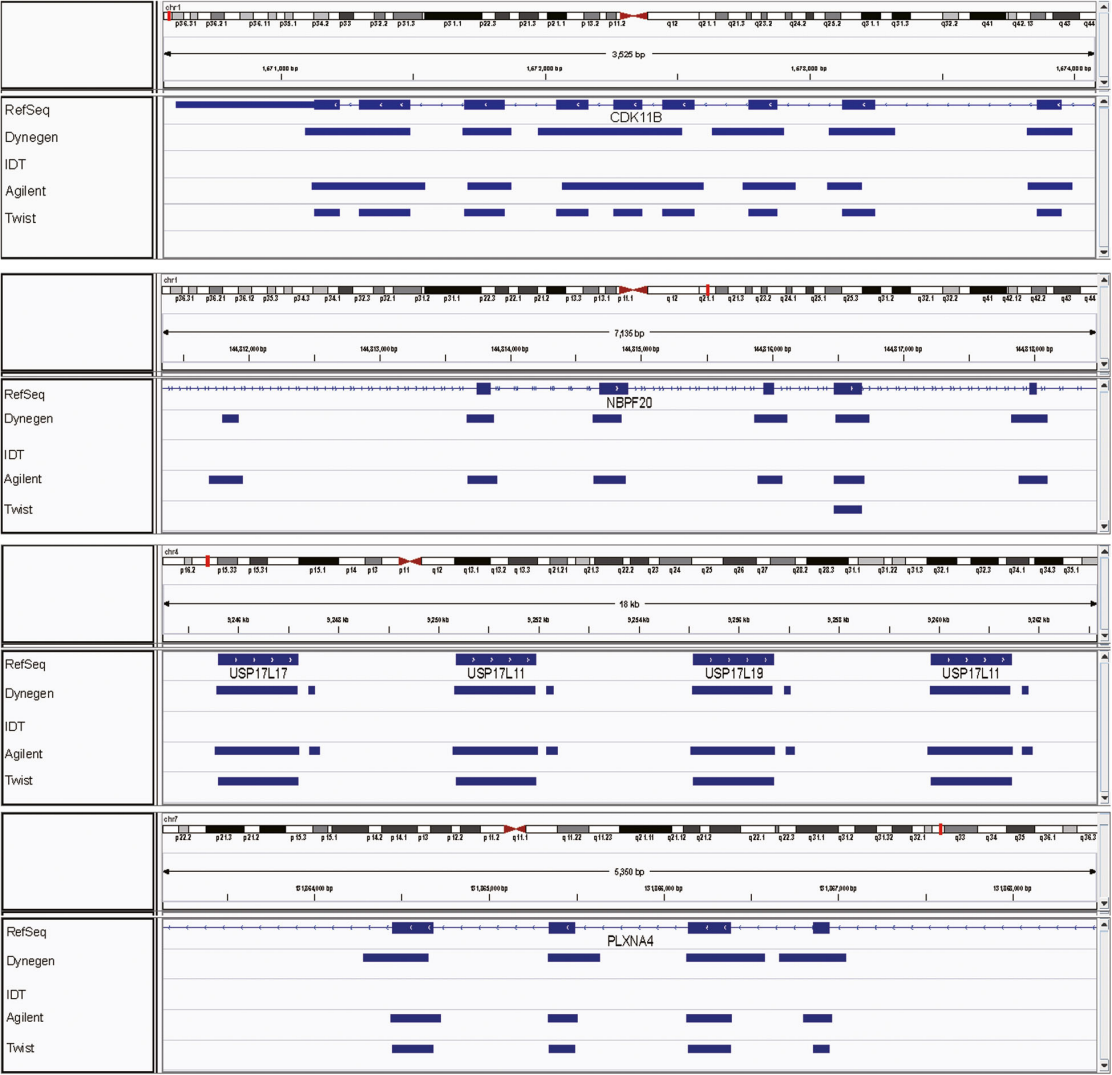
The comparison for the target regions of the four platforms, the blue boxes for RefSeq Genes are exons and for the four *.bed files, it means the region is covered by the panel while loss of the blue boxes means the region is not covered

The differences among the four whole-exome capture platforms with different bait types were shown in Table 1. The target regions and total bait length of the platforms are not identical. For instance, the total bait length of Agilent is 49.48 Mb, Twist is 33.05 Mb, IDT is 50.84 MB and Dynegen is 60.44 Mb. For each platform, we investigated their coverage of RefSeq (35.76 MB), CCDS (32.28 MB), GENCODE.v24 (35.4 MB) and KnownGene (36.75 Mb). Dynegen covers the greatest portion of KnownGene (95.03%) while the other databases are best covered by Agilent. By comparing their specific cover regions, we found that platforms with RNA baits cover greater portion of exome, while platforms with DNA baits, IDT and Twist platforms, mainly focus on the regions of genome easy to capture (Fig. 1). The target region of the platforms for many genes such as *CDK11B*, *NBPF20* and *PLXNA4*, of which, several exons are not covered by the platforms with DNA baits.

**Table 1:** Exome capture technology designs

		Agilent	Twist	IDT	Dynegen
Bait type		ssRNA	dsDNA	ssDNA	dsRNA
Bait length (bp)		120	120	120	120
Total bait length (MB)		49.48	33.05	50.84	60.44
Total target length (MB)		35.7	NP	39.0	NP
Method of library preparation		Dynegen	Twist	Dynegen	Dynegen
Fragmentation method		Ultrasonication	Enzymatic Fragmentation	Ultrasonication	Ultrasonication
DNA input for library preparation (ng)		30	30	30	30
%Exome covered	RefSeq(35.76 Mb)	0.9533	0.9235	0.9357	0.9445
	CCDS(32.28 Mb)	0.9987	0.9967	0.9933	0.9924
	GENCODEv24(35.4 Mb)	0.9943	0.9148	0.9344	0.9753
	KnownGene(36.75 Mb)	0.9430	0.9098	0.9240	0.9503

ds: double stranded; ss: single stranded; NP: not provided

### Target coverage efficiency of the four whole-exome platforms

**Fig. 2:**
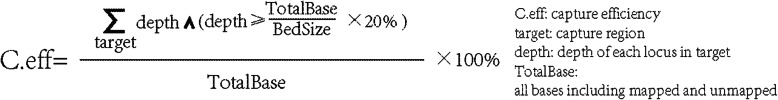
The formula of capture efficiency

We formulate capture efficiency as a novel parameter that can be better used to evaluate the specificity and coverage depth among the different capture platforms. The formula was shown in Fig. 2.

**Fig. 3:**
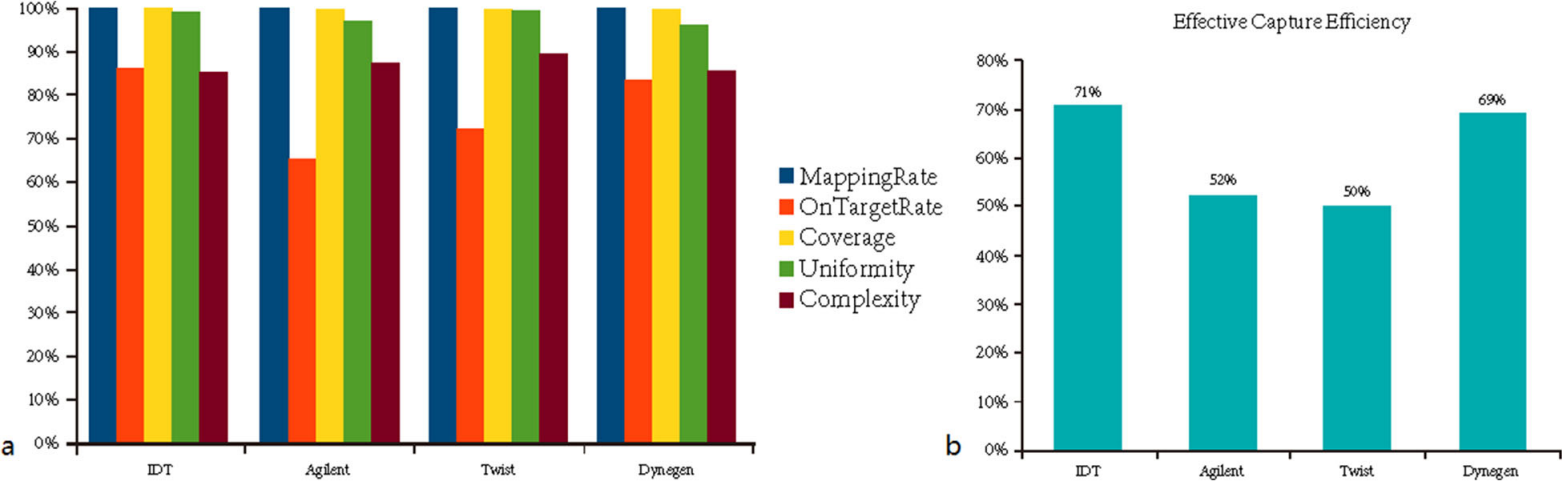
Target coverage efficiency of the four platforms

With the normalized read counts to the theoretical average sequencing depth of 150×, the mapping rate of the four platforms all exceeded 99%. There were differences in actual average depth and on target rates among the four platforms (Fig. 3a). The platform with single stranded DNA baits achieved the highest on target rate (86%), followed by the platform with double stranded RNA baits (83%). The uniformity of the four platforms all exceeded 95% and the platform with double stranded DNA baits reached the highest uniformity of 99.32%, which also achieved the highest complexity of the four platforms (89.26%). The capture efficiency is calculated separately for the four platforms. The platform IDT, with single stranded DNA baits, achieved the highest capture efficiency of the four platforms (71%), followed by the platform Dynegen with double stranded RNA baits (69%) (Fig. 3b).

**Fig. 4:**
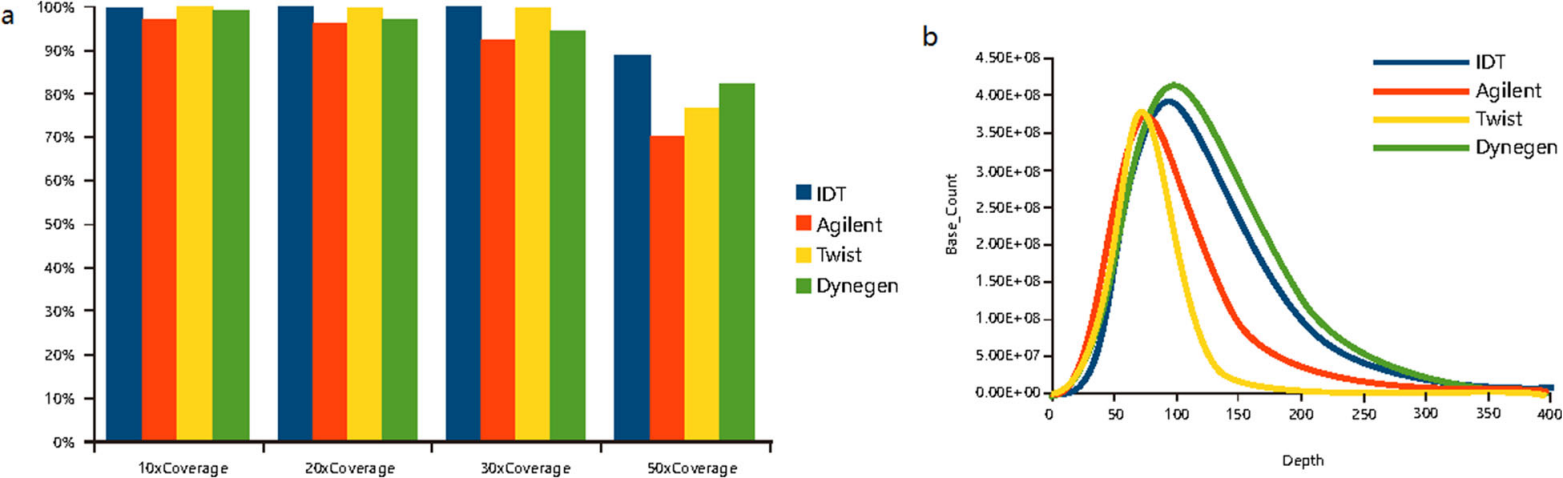
The fraction of the target bases covered at different depths

We also determined the fraction of the target bases covered at depths of at least 10×, 20×, 30× and 50× (Fig. 4a). All the platforms covered about 95% of target bases with depth of at least 20×. For depth of at least 30×, platforms with DNA baits covered higher fraction of the target bases than other two platforms. Based on the cumulative depth distribution curve (Fig. 4b), we also found that the platform with double stranded RNA baits, Dynegen, covered more bases with ≥100× coverage. In addition, we compared the relationship between the actual average sequencing depth and 10×, 20×, 30×, 50× coverage for each replicate of each platform. The results were shown in Fig. S1. We found no major difference in coverage efficiency among four technical replicates, indicating that all four platforms give high technical reproducibility, and the proportion of target regions with high coverage depth is positively correlated with the actual average sequencing depth. The target coverage efficiency results for each replicate are added in Table S1.

### Influence of GC content on coverage

**Fig. 5:**
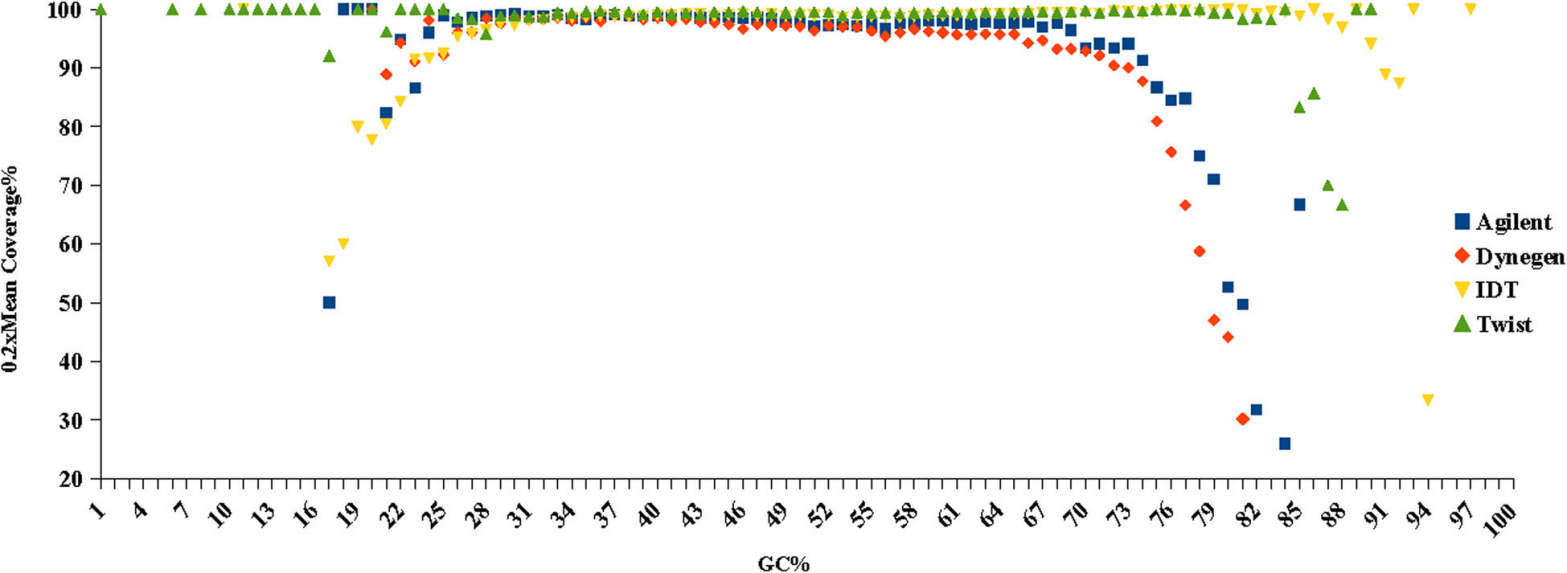
The proportions of target region with coverage of more than 20% average depth at different GC contents for the four platforms

To study the GC bias effect, we evaluated the reads count at different GC content and plotted GC content against the depth distribution for the four platforms (Figs. S2 and S3). The average GC content of the normalized data set for the platforms with DNA baits (50.46% for IDT, 50.84% for Twist) is higher than the platforms with RNA baits (48.46% for Agilent, 48.26% for Dynegen). With different GC content, the proportions of target region with coverage of more than 20% of the average depth for the four platforms are shown in Fig. 5. These four platforms showed a similar uniformity from 25% GC content to 70% GC content. For higher GC content, the DNA baits showed better uniformity. However, many high GC regions of genes have been not included by the covered bed file of DNA baits platforms.

**Fig. 6:**
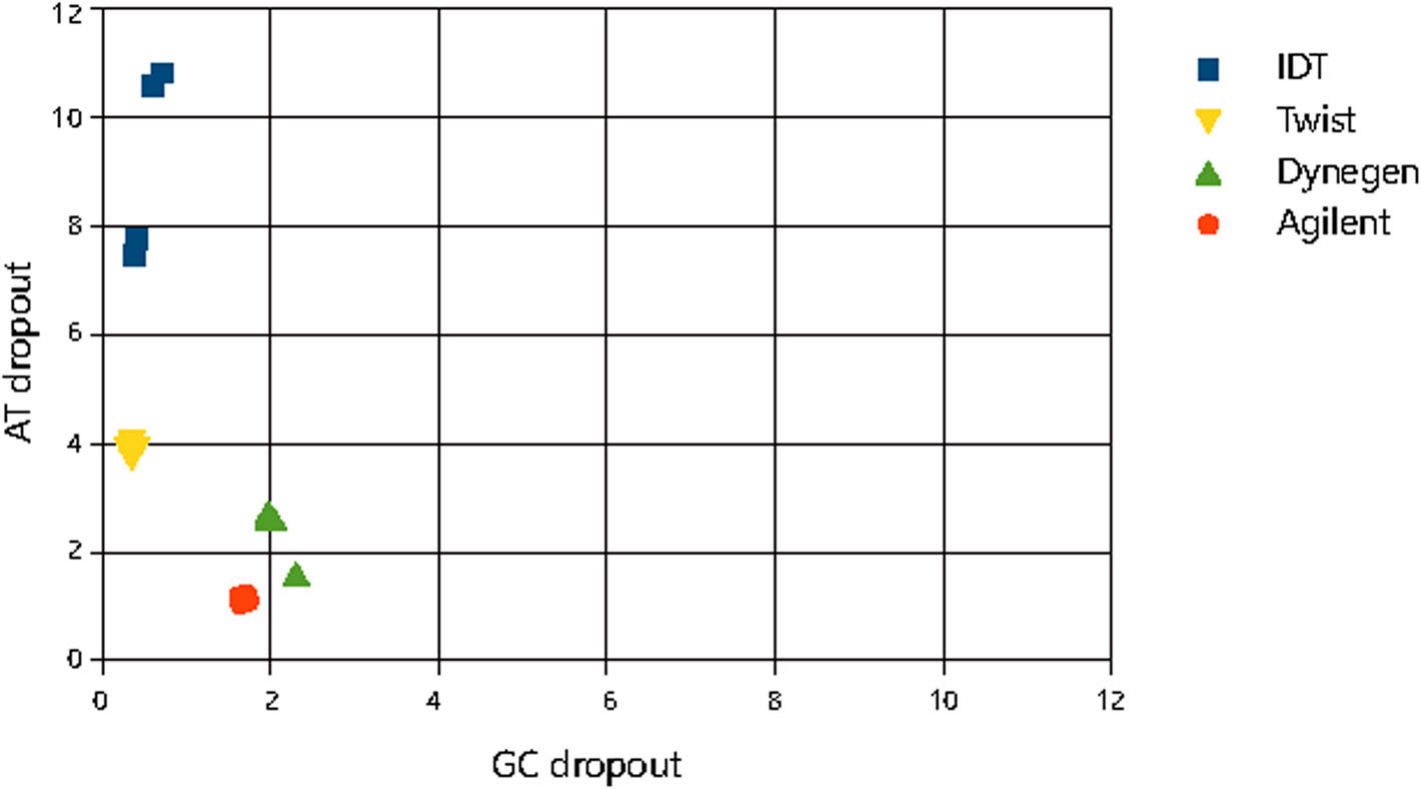
AT/GC dropout for the four platforms

In addition, we also calculated AT/GC dropout which shows the fraction of total reads that should have mapped to GC > 50% or GC < 50% regions mapped elsewhere. The results of AT/GC dropout were shown in Fig. 6. AT/CG dropout of platforms with RNA baits are very low, but for platforms with DNA baits, although their GC dropouts are low, AT dropout for them are high, especially for platform with single stranded DNA, AT dropout up to 10%. All the results reveal that different types of baits may have capture bias for regions with different characteristics.

### Ability to detect SNPs and Indels

The aim of whole-exome resequencing is to identify variants. Therefore, we systematically compared the ability to detect SNPs and Indels among the four exome capture platforms. When considering the variants identified in their respective target regions, there is a clear correlation between the total number of SNPs and Indels detected and the number of bases targeted. The platform with double stranded RNA baits and the largest target region, Dynegen, detected the highest number of SNPs and Indels followed by IDT and Agilent which is shown in Table 2. As well as the platform with double stranded DNA baits and the smallest target region, Twist platform detected the fewest SNPs and Indels. We also compared SNPs detected in our study with the known SNPs of NA12878 and dbsnp138. As result, the frequency of SNP concordance to NA12878 and dbsnp138 on target for the four platforms are all more than 85 and 95%. And Dynegen platform with double stranded RNA baits detected the greatest number of SNPs concordance to NA12878 and dbsnp138. All the results are shown in the Table 2. In addition, the ratio of the count of Indels on target to total bait length of Twist is significantly lower than the other platforms.

**Table 2:** SNPs and Indels detection

Sequencing QC Metric	IDT	Agilent	Twist	Dynegen
Sample ID	S074_5	9	A298	A1
Percent Selection	48	55	32	63
Percent Duplication	15.09	12.93	10.74	14.7
Estimated Library Size (millions)	5794.4	5863.3	3796.4	7050.5
Count SNP on Target	40,729	41,748	22,964	54,340
Count INDEL on Target	5154	4852	806	6603
SNP concordance to dbsnp138 on Target	99.00%	97.00%	98.00%	97.00%
SNP concordance to NA12878 on Target	90.00%	85.00%	88.00%	87.00%
Sensitivity 2 HET on Target referring dbsnp138	99.00%	95.00%	97.00%	96.00%
Percent A < --->G Count on Target	36.04	35.23	37.15	35.19
Percent C < --->T Count on Target	35.81	35.91	37.40	35.27
Percent INS/INDEL on Target	52.20	50.90	50.00	51.37
Percent INDEL (length = 1) on Target	58.67	61.29	28.78	61.00
Percent INDEL (length < =10) on Target	93.69	94.87	84.24	94.99

We also investigated whether these four platforms showed bias in substitution detection, and none of them showed bias. The transition-transversion (ts/tv) ratio ranged from 2.38 in Dynegen to 2.93 in Twist. Previous studies have shown ts/tv ratios of 2.0 ~ 2.1 for whole genome datasets [6]. The difference in ts/tv ratios among the four platforms may be caused by their different target regions.

**Fig. 7:**
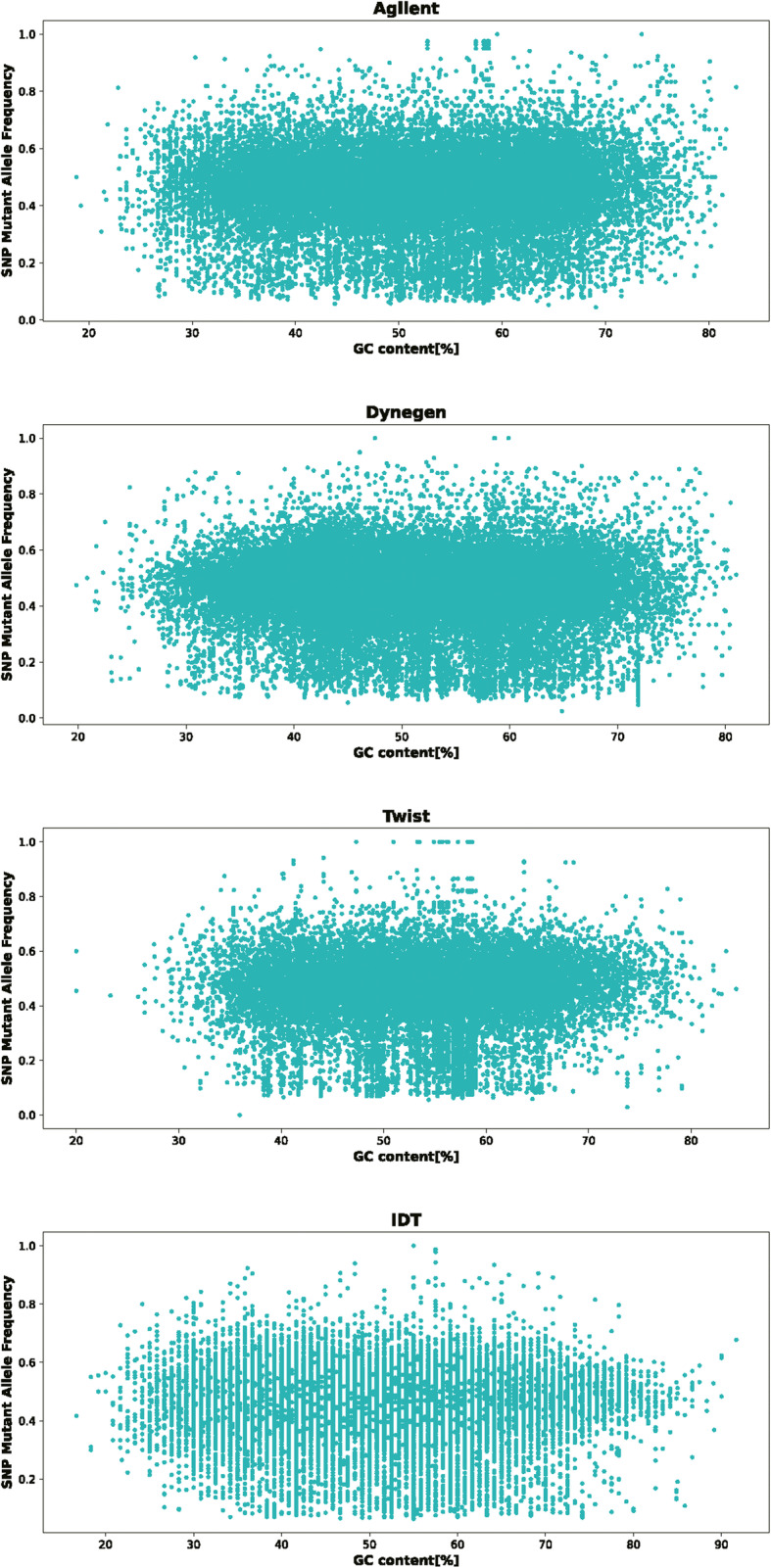
The frequency distribution of heterozygous SNPs at different GC content

**Fig. 8:**
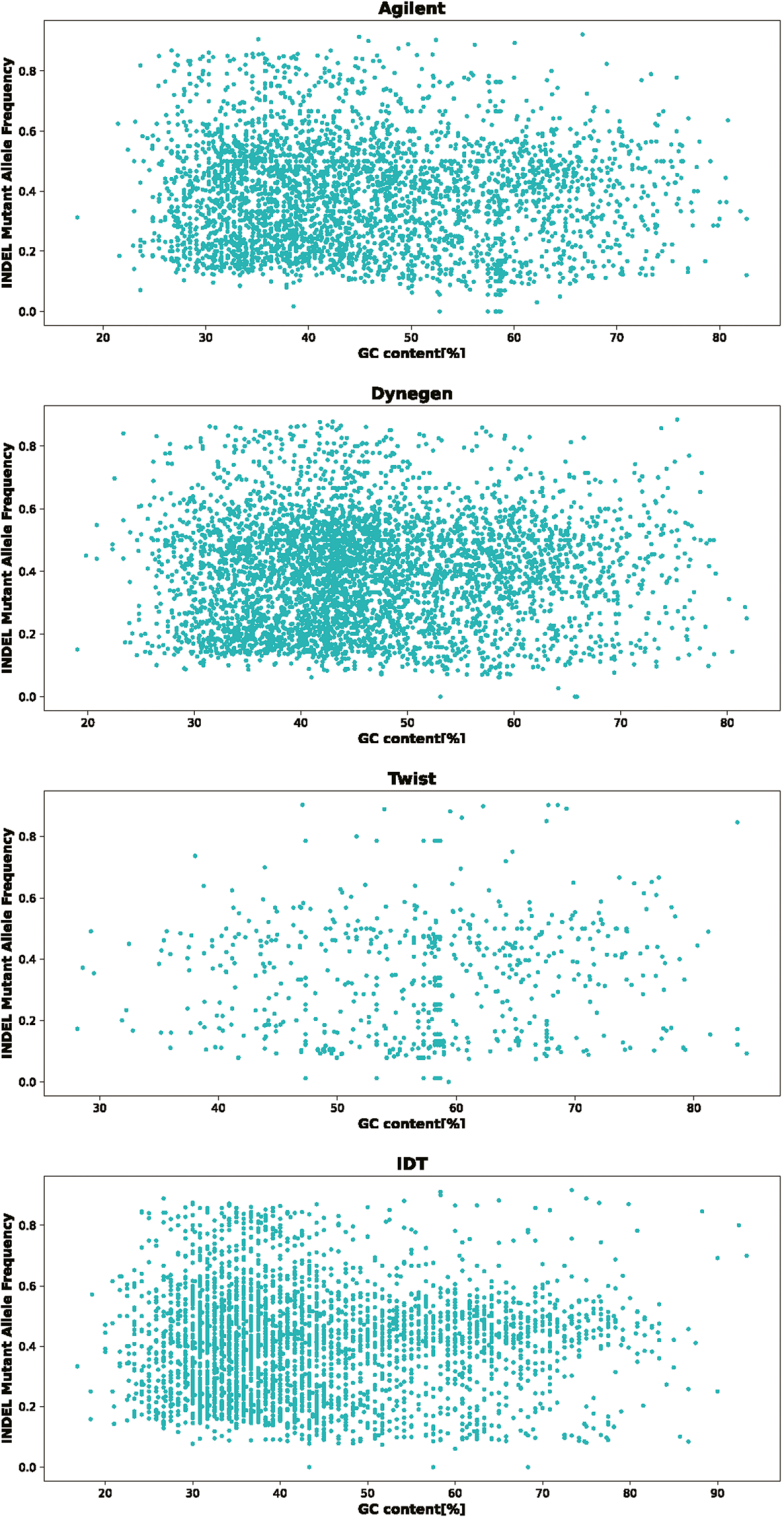
The frequency distribution of heterozygous Indels at different GC content

Approximately half of the Indels are on target for all four platforms. Except for the Twist platform, more than 90% of the Indels less than ten bases length are on target for the other platforms. We also analyzed the frequency distribution of heterozygous SNPs/Indels at different GC content and the results are shown in Figs. 7 and 8. We calculated the dispersion degree of the heterozygous SNPs/Indels frequency distribution according to the Student’s t distribution and the difference between the upper and lower boundaries of 95% confidence interval was used to indicate dispersion degree [7]. We found that IDT and Dynegen have the smallest dispersion of heterozygous SNPs/Indels frequency distribution.

### Performance comparison of RNA baits with ultra deep sequencing

**Fig. 9:**
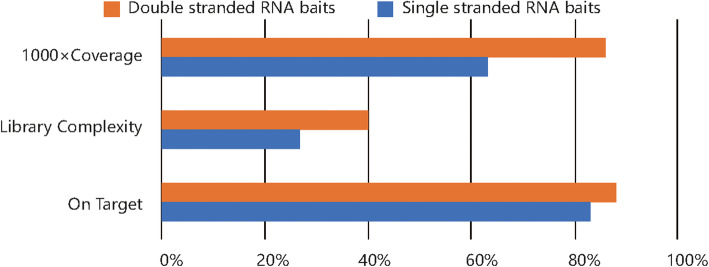
ctDNA detection performance between single stranded baits and double stranded baits

We further compared the capture performance of the platforms with single or double stranded RNA baits at ultra deep sequencing. The comparison of on target rate, > 1000× coverage and complexity for each baits type were conducted and the results were shown in Fig. 9. Double stranded RNA baits of Dynegen platform perform better than single stranded RNA baits of Agilent platform in all three aspects. According to our results, double stranded RNA probes can capture more information from the library constructed by very low input samples with ultra deep sequencing, which suggesting that double stranded RNA baits may improve the mutation detection sensitivity in liquid biopsy applications.

## Discussion

We present a comparative study of four whole-exome capture platforms with different types of capture baits from several important aspects. Four parameters for each whole-exome platform were analyzed: the designed features of each whole-exome panel, target coverage efficiency, GC bias and sensitivity in SNPs/Indels detection. We also first defined capture efficiency which calculates the proportion of data on target with more than 20% average depth in the total data. Therefore, it reflects the effective utilization of sequencing data for each platform better and avoids some false negative of variants detection in the data set with high on target rate but low coverage depth.

All the four exome capture platforms cover large portions, exceeding 99%, of the CCDS exome. The two platforms with RNA baits cover larger portions of all the four databases than the other two platforms with DNA baits. The regions difficult to capture are not covered by the platforms with DNA baits. Early comparisons of whole-exome capture platforms reported that at a minimal depth of 20×, all platforms were able to cover around 80% of the targeted regions [8, 9]. In our study, the four platforms all cover over 95% of their targeted regions at minimal depth of 20×. The platforms with double stranded RNA or single stranded DNA achieve the highest on target rate and capture efficiency. At a minimal depth of 100×, the platform with double stranded RNA baits captures the largest proportion of the target region among the four platforms. Although the capture efficiency of the platform with DNA baits, IDT platform, is a little higher than Dynegen platform with RNA baits, considering the target region covered by the panel respectively and the higher capture efficiency of the platform with RNA baits, Agilent platform, than Twist platform with DNA baits, we infer that RNA baits have stronger binding power than DNA baits and single or double stranded bait types may affect capture performance. So we further compared the capture performance of single and double stranded RNA baits with the same custom designed panel and ultra deep sequencing. All the results show that the performance of double stranded RNA baits is better than single stranded suggesting that with ultra deep sequencing, double stranded RNA baits may improve the mutation detection sensitivity especially for trace samples with ultra low mutation frequency.

Although all the four platforms showed the bias against very low and high GC content, the platforms with DNA baits (single stranded DNA baits and double stranded DNA baits) perform better in high GC content region. The platforms with RNA baits (single stranded RNA baits and double stranded RNA baits) perform better in high AT content region. This may be caused by high or low GC content reducing the efficiency of polymerase chain reaction (PCR) amplification [10] and efficiency of capture probe hybridization [11]. AT dropout and GC dropout of Dynegen are both about 2%, while AT dropout of IDT is about 8% and GC dropout is less than 1%. These different performances of the platforms may be caused by their different bait types. In the future, mixed probes of double stranded RNA and single stranded DNA may be used in target capture in order to get the potential better capture effect from high AT region to high GC region.

We also compared the sensitivity in SNPs/Indels detection for the four platforms. Dynegen detected the most SNPs and Indels in the sample of NA12878. SNP concordance to dbsnp138 is 97% and SNP concordance to NA12878 on target is 87%. Twist detected the minimum number of SNPs and Indels which may be caused by its smallest target capture regions. The results suggested that Dynegen is the better choice to identify novel variants and Twist is better to detect the known disease-causing mutations with the minimum amount of sequencing data. The capture efficiency of the platforms may seem high because of the design of the capture panel. But some important variants may still fail to be detected, which cannot meet the needs of scientific research. Therefore, the difficult-to-capture regions should not be intentionally excluded just because of the better capture efficiency.

However, there are still some limitations to our study. Because the workflow of library construction for Twist platform is very different from other platforms, and we hope to test the performance of the entire set of Twist reagents kits, library construction kits from Dynegen Bioscience were not used for Twist platform. Other platforms with different baits types all use the same fragmentation methods and the choice of library construction kits is very flexible. Therefore, we chose library construction kits from Dynegen Bioscience which has high efficiency from original sample converted to library. Because of the limitation of reagents and funds, we only performed four technical replicates for each platform. When comparing the differences between Twist platform and other platforms, it may be difficult to visually prove that the differences are caused by the capture baits types or the different library construction kits. Too small sample sizes will also cause insufficient statistical power. It is difficult to verify that the differences among the groups come from systematic errors or the different types of capture baits.

Next-generation sequencing technologies evolve rapidly, and each exome capture platform is still updating. For instance, the platform with double stranded RNA, Dynegen platform, recently released the new version of QuarXeq Human All Exon Probes which not only covers the exome regions but also covers most SNP loci in the whole genome. Meanwhile, the operation flow of library preparation is further simplified, greatly reducing the experiment time. In this study, we found that RNA baits may have stronger binding power than DNA baits, and the binding power may be different because of the single or double stranded bait types. In addition, the binding power of DNA and RNA baits to genome regions with different characteristics is different. Therefore, in the future, mixed probes of double stranded RNA and single stranded DNA may be used in target capture to get the potential better capture effect.

## Conclusions

The target capture platforms with double stranded RNA baits have the most balanced capture performance. Especially with ultra deep sequencing, platform with double stranded RNA baits may improve the sensitivity of ultra low frequent mutation detection. Our results also show that the binding power of RNA and DNA baits to different genome regions is different, so we propose that the mixed baits of double stranded RNA and single stranded DNA may improve target capture performance.

## Methods

### DNA sample

DNA sample of HapMap-CEPH NA12878 was obtained from Coriell. NA12878 is collected from a Utah female. It has been genotyped in the International HapMap project, and repeatedly sequenced by different platforms of next-generation sequencing. Therefore, it is the most thoroughly studied human diploid genome.

Multiplex I cfDNA Reference Standard Set obtained from Horizon. The sample set is derived from human cell lines. It is highly-characterized, biologically-relevant reference materials used to assess the performance of cfDNA assays that detect somatic mutations. There are four samples in each Multiplex I cfDNA Reference Standard Set covering eight engineered single nucleotide variants, involving four genes of *EGFR*, *KRAS*, *NRAS*, and *PI3KCA*, at 5%, 1%, and 0.1% allelic frequencies respectively.

### Library preparation, target capture and next-generation sequencing

**Fig. 10:**
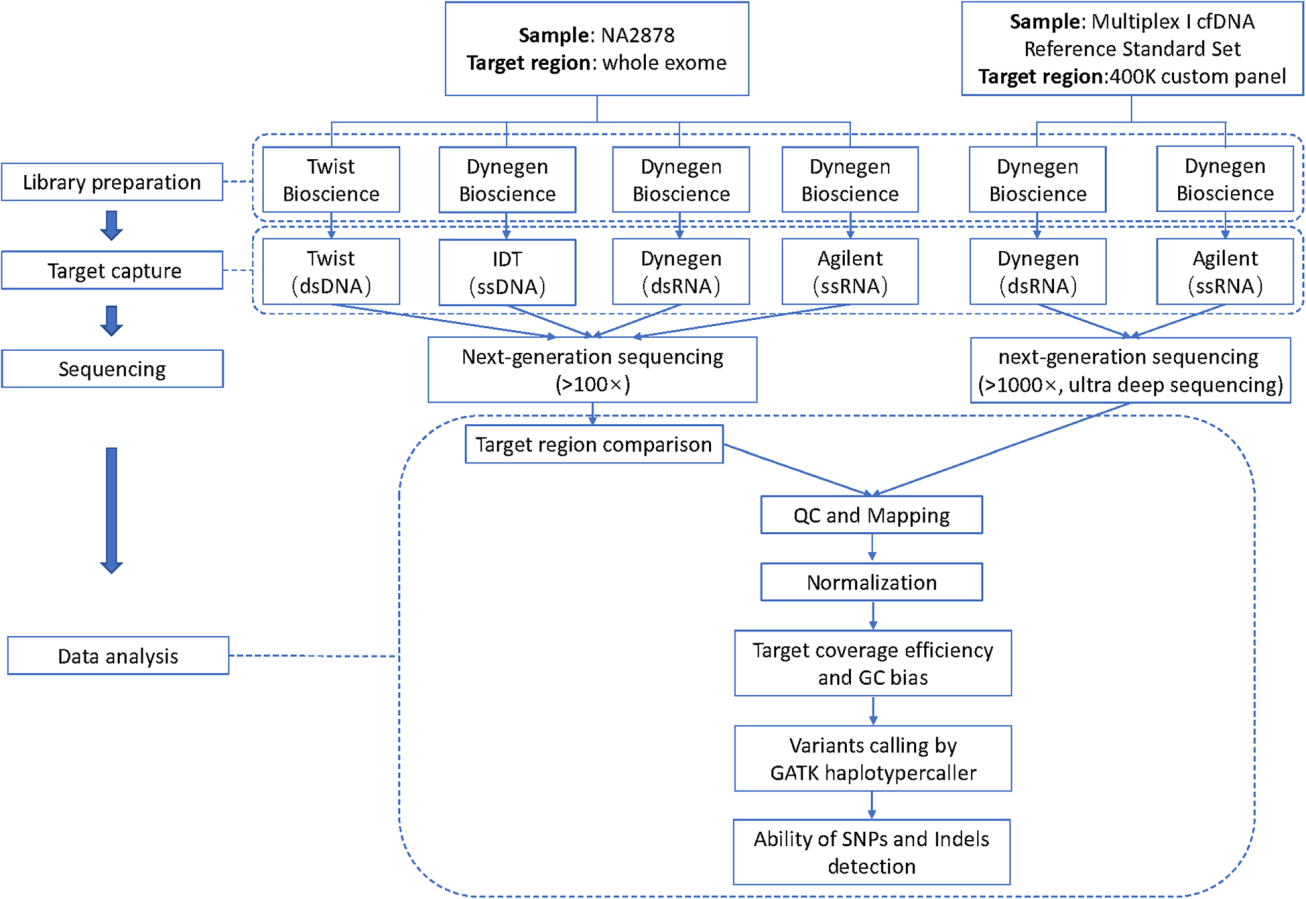
The design and workflow of the study

DNA sample of NA12878 was used to produce each whole-exome captured sequencing library for the platforms of Agilent Technologies with single stranded RNA baits, Integrated DNA Technologies (IDT) with single stranded DNA baits, Twist Bioscience with double stranded DNA baits and Dynegen Bioscience with double stranded RNA baits. The manufacturers’ protocols of Human Core Exome (Twist Bioscience) and QuarXeq Human All Exon Probes (Dynegen Bioscience) were used for library preparation and exome capture on Twist Bioscience and Dynegen Bioscience platform respectively. For the platform of Agilent Technologies and IDT, Library construction was performed with the reagents and protocol supported by Dynegen Bioscience as well as exome capture were performed with the reagent kits and manufacturer’s protocols of SureSelect All Exon v7 and xGEN Exome Research Panel v1.0 respectively. The design and workflow of the study were shown in Fig. 10 and the size distribution of the libraries is shown in Fig. S4.

The samples of Multiplex I cfDNA Reference Standard Set were used to produce the custom target captured library for the capture platforms with single and double stranded RNA respectively. The custom panel is about 400 KB covering 85 genes related to biliary tract cancer synthesized by Agilent Technologies and Dynegen Bioscience respectively. 30 ng input standard cfDNA and KAPA HyperPrep Kits were used for library construction and the library was divided into two parts for capture using single and double stranded RNA baits respectively. Ultra deep sequencing was conducted for each custom post-captured library.

The pre-captured and target captured sequencing libraries were quality-controlled using 2100 Bioanalyzer with High Sensitivity DNA kits (Agilent Technologies, United States) and Qubit™ 3.0 Fluorometer with Qubit™ dsDNA HS Assay Kit (Invitrogen, ThermoFisher Scientific, United States). The Illumina HiSeq X Ten System (Illumina, United States) was used to sequence the final DNA libraries as PE 150 bp reads.

### Data analysis

The human reference genome (hg19), RefSeq, CCDS, Ensembl and KnownGene databases were downloaded from the UCSC genome table browser (http://genome.ucsc.edu/). The coverage of the four different exome capture platforms to the public databases was calculated.

FastQC tool was used for quality control of the initial FASTQ files. Trimmomatic was used to remove adapters. Burrows-Wheeler Aligner (BWA) [12] was used to align raw reads to the human reference genome (hg19). Genome Analysis Toolkit (GATK) Best Practices, the pipeline for germline short variant discovery, was run on every data set independently. GATK haplotypercaller was used to call SNPs and InDels.

We normalized read counts of each whole-exome data set by randomly drawing reads to the theoretical average sequencing depth of 150× and custom captured sequencing data set of 5000× using Picard’s DownsampleSam. Mapping rate, on target rate, coverage, uniformity, complexity and capture efficiency were calculated. The relationship between the average sequencing depth and 10×, 20×, 30×, 50× coverage of each normalized whole-exome data set was compared and the cumulative depth distribution curve was plotted.

Picard CollectGcBiasMetrics was used to calculate the overall GC distribution. Picard CollectHsMetrics was used to calculate the AT/GC dropout of each sample. The relationship between GC% and depth was analyzed by Python, and RStudio was used for graphing.

## Supplementary Information

**Additional file 1.** Table S1.

**Additional file 2.** Fig.S1 The relationship between the average sequencing depth and the proportion of target region with exceeding 10×, 20×, 30×, 50× coverage for the four platforms.

**Additional file 3.** Fig.S2 Reads count distribution against different GC content for the four platforms.

**Additional file 4.** Fig.S3 GC content against the depth distribution for 4 platforms.

**Additional file 5.** Fig.S4 The size distribution of the libraries. DY-V7 is the size distribution of the library constructed by Agilent platform; Twist-L is the size distribution of the library constructed by Twist platform.

## Data Availability

All data generated or analysed during this study are included in this published article and its supplementary information files.

## Abbreviations

SNPs: single nucleotide polymorphisms
Indels: small insertions and deletions
ts/tv: transition-transversion ratio
PCR: polymerase chain reaction
IDT: Integrated DNA Technologies
GATK: Genome Analysis Toolkit
BWA: Burrows-Wheeler Aligner
