# Correction to: Performance comparison of four types of target enrichment baits for exome DNA sequencing

**DOI:** 10.1186/s41065-023-00296-7

**Published:** 2023-09-06

**Authors:** Juan Zhou, Mancang Zhang, Xiaoqi Li, Zhuo Wang, Dun Pan, Yongyong Shi

**Affiliations:** Bio-X Institutes, Key Laboratory for the Genetics of Developmental and Neuropsychiatric Disorders (Ministry of Education), Collaborative Innovation Center for Brain Science, Shanghai Jiao Tong University, Shanghai, 200030 People’s Republic of China

**Correction: Hereditas (2021) 158:10**

10.1186/s41065-021-00171-3

Following the publication of the original article [1], the competing interest section will be updated to:

## Competing interests

Dr. Yongyong Shi is a Chief Scientist for Dynegene and a Co-Chief Editor for Hereditas. All the other authors declare that they have no competing interests.
