# Supplementary figures and images for: Performance comparison of four types of target enrichment baits for exome DNA sequencing
Source: Hereditas. 2021 Feb 17;158:10. doi: 10.1186/s41065-021-00171-3 (PMC7888174; doi:10.1186/s41065-021-00171-3)

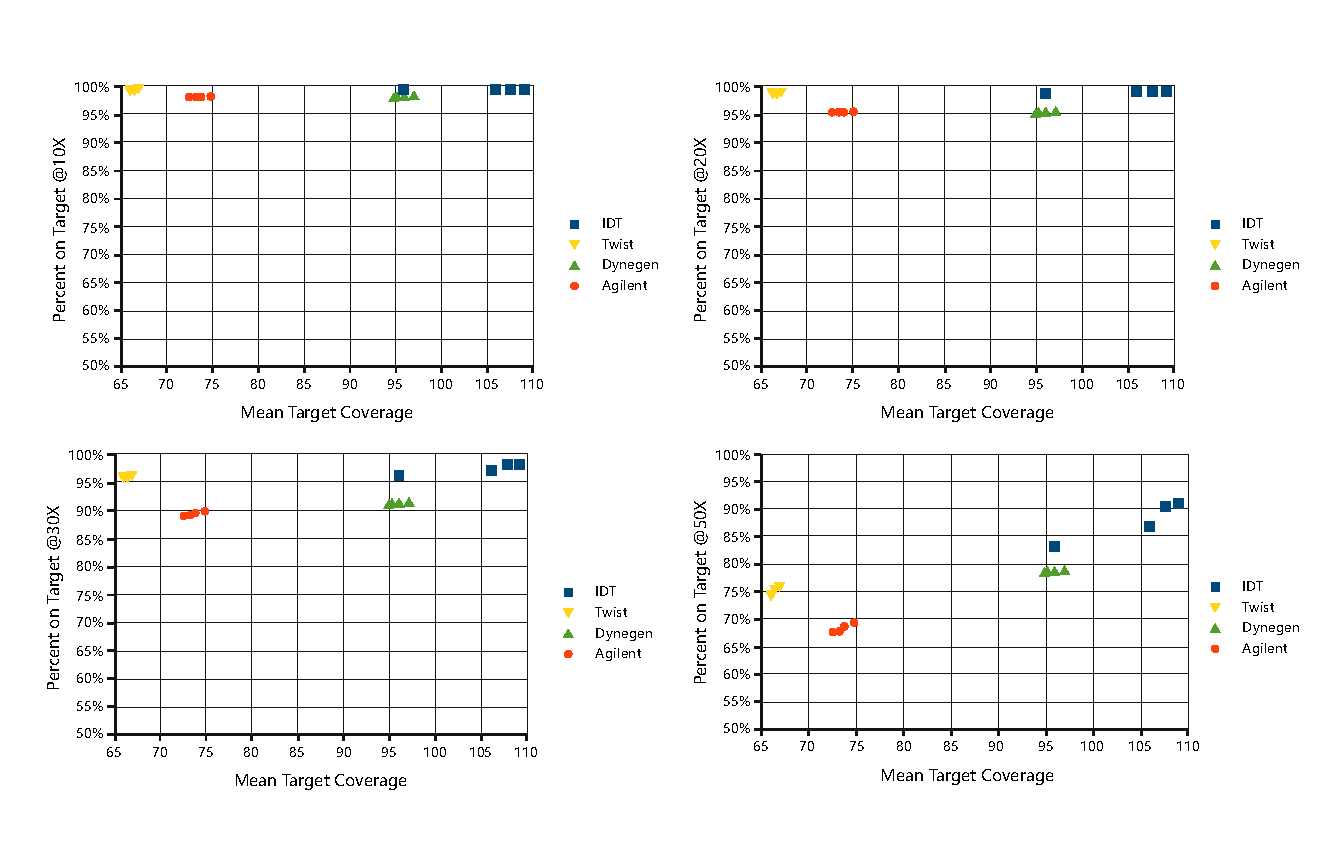

Supplement: Supplementary file 2 — Additional file 2. Fig.S1 The relationship between the average sequencing depth and the proportion of target region with exceeding 10×, 20×, 30×, 50× coverage for the four platforms. [file 41065_2021_171_MOESM2_ESM.tif]

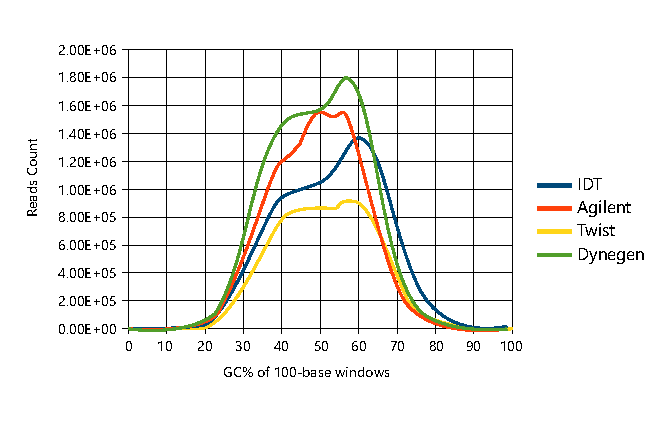

Supplement: Supplementary file 3 — Additional file 3. Fig.S2 Reads count distribution against different GC content for the four platforms. [file 41065_2021_171_MOESM3_ESM.tif]

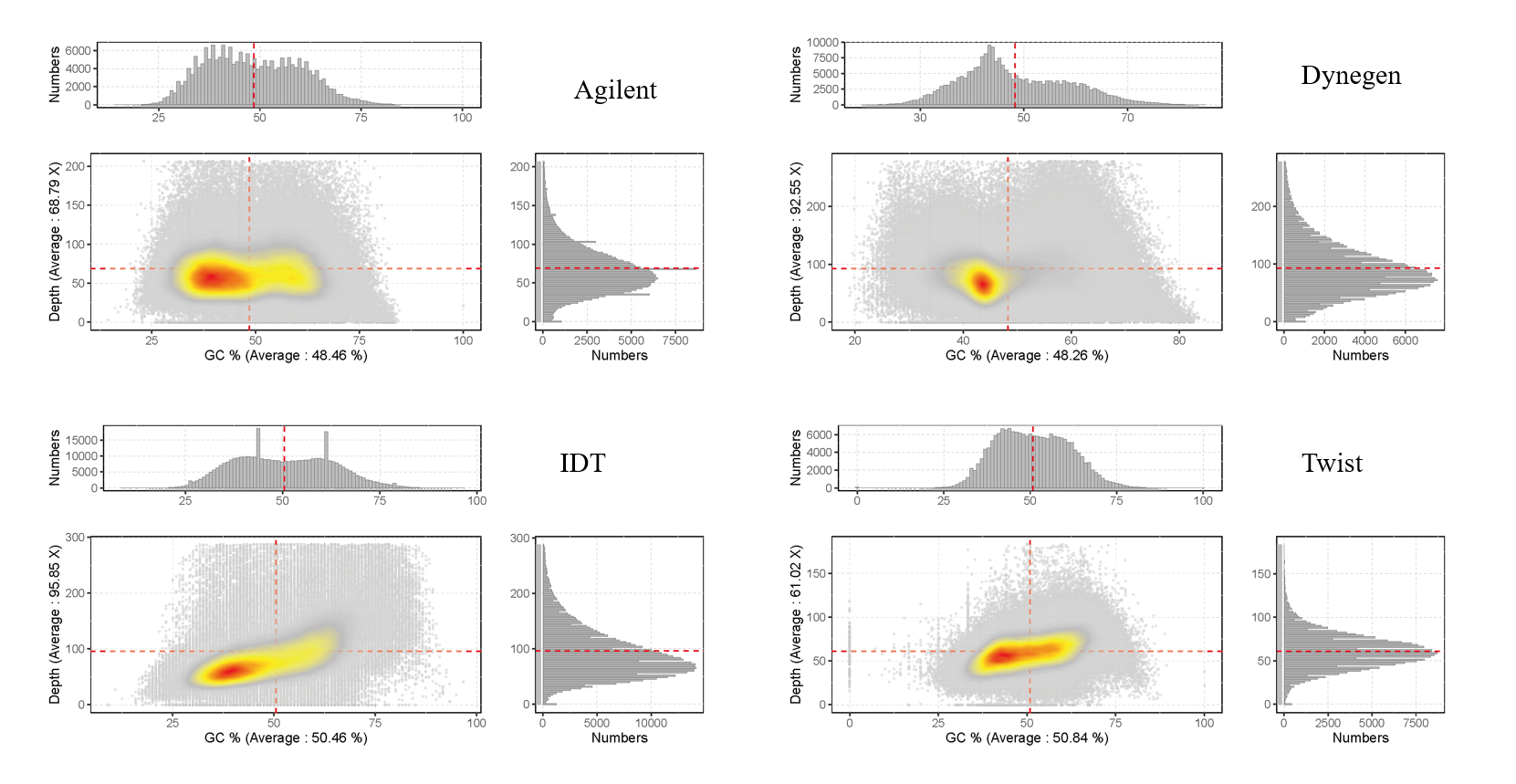

Supplement: Supplementary file 4 — Additional file 4. Fig.S3 GC content against the depth distribution for 4 platforms. [file 41065_2021_171_MOESM4_ESM.tif]

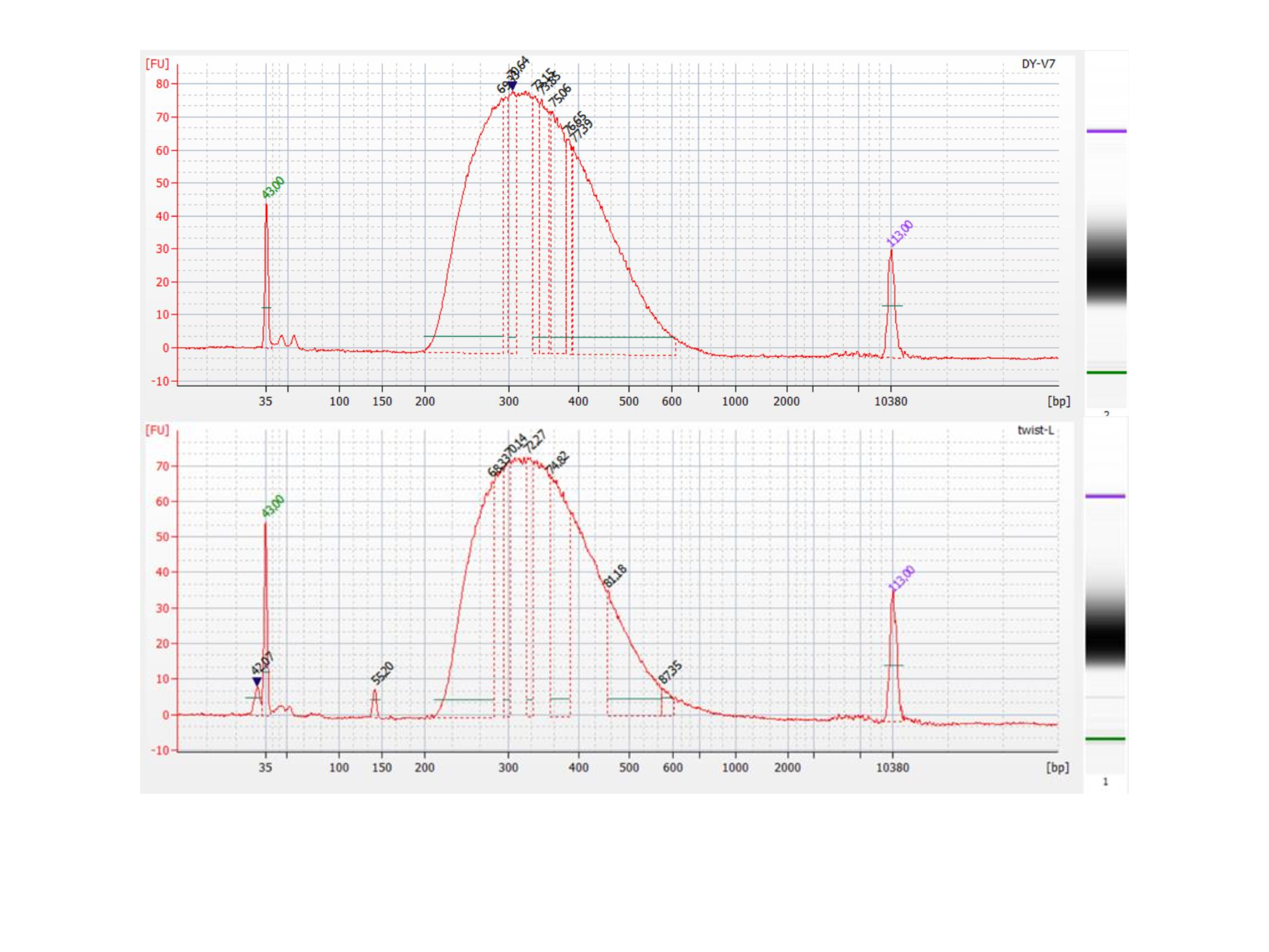

Supplement: Supplementary file 5 — Additional file 5. Fig.S4 The size distribution of the libraries. DY-V7 is the size distribution of the library constructed by Agilent platform; Twist-L is the size distribution of the library constructed by Twist platform. [file 41065_2021_171_MOESM5_ESM.tiff]
